# Antibiotic prescribing in women during and after delivery in a non-teaching, tertiary care hospital in Ujjain, India: a prospective cross-sectional study

**DOI:** 10.1186/2052-3211-6-9

**Published:** 2013-11-04

**Authors:** Megha Sharma, Linda Sanneving, Kalpana Mahadik, Michele Santacatterina, Suryaprakash Dhaneria, Cecilia Stålsby Lundborg

**Affiliations:** 1 Global Health, IHCAR, Department of Public Health Sciences, Karolinska Institutet, SE 171 77, Stockholm, Sweden; 2 Department of Pharmacology, R. D. Gardi Medical College, Ujjain, (M P) 456010, India; 3 Department of Obstetrics and Gynecology, R. D. Gardi Medical College, Ujjain, (M P) 456010, India

**Keywords:** Antibiotic prescribing, Vaginal delivery, Cesarean section, Non-teaching hospital, Ujjain, Madhya Pradesh, India

## Abstract

**Objectives:**

Antibacterial drugs (hereafter referred to as antibiotics) are crucial to treat infections during delivery and postpartum period to reduce maternal mortality. Institutional deliveries have the potential to save lives of many women but extensive use of antibiotics, add to the development and spread of antibiotic resistance. The aim of this study was to present antibiotic prescribing among inpatients during and after delivery in a non-teaching, tertiary care hospital in the city of Ujjain, Madhya Pradesh, India.

**Methods:**

A prospective cross-sectional study was conducted including women having had either a vaginal delivery or a cesarean section in the hospital. Trained nursing staff collected the data on daily bases, using a specific form attached to each patient file. Statistical analysis, including bivariate and multivariable logistic regression was conducted.

**Results:**

Of the total 1077 women, 566 (53%) had a vaginal delivery and 511 (47%) had a cesarean section. Eighty-seven percent of the women that had a vaginal delivery and 98% of the women having a cesarean section were prescribed antibiotics. The mean number of days on antibiotics in hospital for the women with a vaginal delivery was 3.1 (±1.7) and for the women with cesarean section was 6.0 (±2.5). Twenty-eight percent of both the women with vaginal deliveries and the women with cesarean sections were prescribed antibiotics at discharge. The most commonly prescribed antibiotic group in the hospital for both the women that had a vaginal delivery and the women that had a cesarean section were third-generation cephalosporins (J01DD). The total number of defined daily doses (DDD) per100 bed days for women that had a vaginal delivery was 101, and 127 for women that had a cesarean section.

**Conclusions:**

The high percentage of women having had a vaginal delivery that received antibiotics and the deviation from recommendation for cesarean section in the hospital is a cause of concern. Improved maternal health and rational use of antibiotics are intertwined. Specific policy and guidelines on how to prescribe antibiotics during delivery at health care facilities are needed. Additionally, monitoring system of antibiotic prescribing and resistance needs to be developed and implemented.

## Introduction

The availability of antibacterial drugs (hereafter referred to as antibiotics) to treat infections during delivery and postpartum period is crucial to reduce maternal mortality. Each year some estimated 350 000 maternal deaths occur worldwide. One of the leading causes of maternal mortality is the infections [1,2]. Infections are estimated by the WHO to be the direct cause of 15% of the global maternal mortality [3], other studies have estimated infections to be the cause of death in as many as 30% of the global maternal mortality cases [4]. The nation-wide Maternal mortality rate (MMR) in India dropped substantially from 570 to 230 per 100 000 live births between 1990 and 2008. However, the overall average pace of the decline in MMR indicates that India will not reach the Millennium Development Goal (MDG) of 108 in 2015 [5]. Recent estimates predicts the MMR will be around 135 by 2015 [2]. A majority of all maternal deaths worldwide occur during delivery and the postnatal period. One of the single most important interventions to reduce maternal mortality is to increase the access to emergency obstetric care [6], which in many cases are dependent on access to antibiotics.

Preventing maternal deaths caused by infections calls for increased access to health care interventions, including access to antibiotics [6]. However, the emerging challenge of antibiotic resistance also calls for precaution on how and when antibiotics are prescribed [7]. In India, there is a widespread use of antibiotics due to both antibiotics being easily available without a prescription and high prescribing rates among health practitioners. Studies conducted at primary and secondary health care facilities in India have shown higher rates of antibiotic prescribing [8-10]. Prescription rates of antibiotics during and after delivery are not well known in the Indian context but there are likely to be cases of both over- and under prescribing.

The aim of this study was to present the prevalence, types and duration of antibiotic prescribed to women during and after vaginal delivery or caesarean section in a tertiary care hospital in the city of Ujjain, Madhya Pradesh, India.

## Methods

### Setting

Ujjain is situated in Madhya Pradesh, which is one of the larger states of India, both in terms of geographical area and in terms of the size of the population. Maternal health indictors for Madhya Pradesh are among the poorest in India. Data from the district level household and family survey conducted in 2007–2008 show that 47% of deliveries take place at a health facility, ranging from 13% in Dindori district to 79% in Indore district [11]. Sixty-six percent of the women in Madhya Pradesh had experienced at least one complication during delivery, and 41% had experienced post-delivery complications including high fever and abdominal pain [10]. In Ujjain district, where data for this study was collected, 90% of the women were covered with antenatal care and 68% of the women had an institutional delivery [11]. There is no general surveillance system to monitor antibiotic prescribing or antibiotic resistance in Madhya Pradesh. However, studies conducted in Madhya Pradesh [9] and in Ujjain district [12-14] have shown overall high prescribing rates among both outpatients and admitted patients. Neither antibiotic prescribing guidelines in general nor specific guidelines for prescribing of antibiotics during vaginal delivery or for obstetric surgery were available at the hospital at the time of the study.

### Data collection

This study was conducted using a prospective cross-sectional design, with data collection from April 2008 to December 2010 at the VD Gardi Charitable Trust Hospital and Research Centre. The hospital is a non-teaching hospital with 350 beds, located in the city of Ujjain and run by the Ujjain Charitable Trust, a non-profit organization where patients pay nominal charges for consultation and treatment. The hospital equally caters to the urban as well as the rural population living in the villages close to the city. The data used for this study was drawn from a large data set, set up by the research group, on the prescribing of antibiotics at this hospital. Trained nursing staff collected the data on daily bases, using a specific form attached to each patient file. The data collection process has been described in detail earlier [14].

### Data management and analysis

Data was entered using Epi info (version 3.1) and Excel, and the analyses were conducted using SPSS (version 21.0) and Stata (version 12.1), Texas, USA. The main variable, *prescribing of antibiotics*, was analyzed separately for the group of women who had had a vaginal delivery and for the group of women that had had a cesarean section. Descriptive statistics was performed to calculate the total prescribed antibiotics in hospital and at discharge, the mean number of days for which antibiotics were prescribed and prescribing by age group, place of residence and days of stay in the hospital. A bivariate and multivariable logistic regression was conducted for the vaginal deliveries to study the association between the binary outcome antibiotic prescriptions (yes, no) and the following variables: age (18–20, 21–30, > = 31), place of residence (Ujjain city, Nearby city, Villages of Ujjain district, Other districts, Cities of the nearby district, Other district villages) and days of stay in the hospital (1–2, 3–5, >5). The term ‘OR’ has been used for the odds ratio of bivariate and ‘adj. OR’ is used for odds ratio of multivariate logistic regressions in the text and in the tables.

The Anatomical Therapeutic Chemical (ATC) classification system and defined daily dose (DDD) was used to classify the prescribed antibiotic [15]. The ATC system divides the active substances into groups and subgroups and the DDD is the assumed average maintenance dose per day for a drug when used for its main indication in adults. The DDD provides a fixed unit of measurement, independent from e.g. strength and price, which enables research on patterns in the prescribing of drugs. For this study, the total DDD and DDD/100 bed days was used to present the prescribing of antibiotics.

### Ethical approval

The study was approved by the Ethics committee of R.D. Gardi Medical College, Ujjain (41-2/2007).

## Result

In total, 1077 women admitted to the VD Gardi Charitable Trust Hospital, who had delivered in the hospital; either as vaginal delivery or cesarean section, were included in the study. Of these 566 (53%) had a vaginal delivery and 511 (47%) had a cesarean section. In the group of women who had a vaginal delivery 491 women (87%) were prescribed antibiotics and in the group of women who had a cesarean section 503 (98%) were prescribed antibiotics in the hospital. The mean numbers of days on antibiotics in hospital for women with a vaginal delivery were 3.1 (±1.7) and for women with cesarean section it was 6.0 (±2.5) (Table 1). Among women that had a vaginal delivery, patients 31 years and above were less likely [est. adj. OR = 0.31 (0.11-0.85); p-value 0.024] than patients in the category 18–20 years (reference group for variable age) to have been prescribed antibiotics during hospital stay or at discharge. The odds of being prescribed antibiotics for women having a vaginal delivery were three times lower [est. adj. OR = 0.31 (0.17-0.59); p-value <0.001] among residents in the category ‘Nearby city’ compared to patients in the category ‘Ujjain City’(reference group for Residence variable). Further, the group of women admitted to the hospital for 3–5 days were more likely to be prescribed antibiotics [est. adj. OR = 2.22 (1.22; 4.02);p-value 0.009] than women admitted 1–2 days. Statistical significance was not found in among women with category of days of stay >5 (p-value 0.085) (Table 2).

**Table 1:** Overview of antibiotic prescribing among patients with vaginal delivery and cesarean section (N = 1077)

		**Diagnosis**	
**Characteristics**		**Vaginal Delivery 566(%)**	**Cesarean Section 511(%)**
Total antibiotic prescriptions^*a^		491 (87)	503 (98)
Prescribed antibiotics after discharge ^b^		160 (28)	141 (28)
Age ^b^			
	18-20	99 (17)	66 (13)
	21-30	429 (76)	401 (78)
	> = 31	38 (7)	44 (9)
Place of Residence ^b^			
	Ujjain city	269 (48)	270 (53)
	Nearby city	90 (16)	67 (13)
	Villages of Ujjain district	81 (14)	60 (12)
	Other districts	50 (9)	53 (10)
	Cities of the nearby district	32 (6)	25 (5)
	Villages of other districts	44 (8)	36 (7)
Days of hospital stay ^+a^			
	1-2	323 (57)	29 (6)
	3-5	192 (34)	175 (34)
	>5	51 (9)	307 (60)
Days on antibiotics^** a^		3.1 ± 1.74	6.0 ± 2.52

^*^ number and percentages; Chi-Square test. ^**^mean and standard deviations; Kruskal-Wallis equality-of-populations rank test. ^a^P-value < 0.05. ^b^P-value > 0.05.^+^Fisher’s exact test.

**Table 2:** Bivariate and multivariable logistic regression on antibiotics prescriptions among patients with vaginal delivery (N = 566)

		**Bivariate**		Multivariable^*^	
**Characteristics**		**OR (95% CI)**	**p-value**	**adj.OR (95% CI)**	**p-value**
Age	18-20	1			
	21-30	0.67 (0.32;1.4)	0.282	0.7 (0.33;1.49)	0.352
	> = 31	0.28 (0.1;0.76)	0.012	0.31 (0.11;0.85)	0.024
Residence	Ujjain city	1			
	Nearby city	0.33 (0.18;0.6)	0.001	0.31 (0.17;0.59)	<0.001
	Villages of Ujjain district	0.89 (0.4;1.98)	0.78	0.76 (0.34;1.71)	0.504
	Other districts	0.82 (0.32;2.1)	0.676	0.8 (0.31;2.08)	0.644
	Cities of the nearby district	0.78 (0.25;2.4)	0.665	0.81 (0.26;2.52)	0.72
	Villages of other districts	0.71 (0.27;1.82)	0.473	0.71 (0.27;1.86)	0.487
Days of stay ^+a^					
	1-2	1			
	3-5	2.07 (1.16;3.68)	0.014	2.22 (1.22;4.02)	0.009
	>5	8.43 (1.14;62.6)	0.037	2.59 (0.88;7.66)	0.085

^*^ adjusted by place of residence, age and duration of stay.

The most commonly prescribed antibiotic groups during the hospital stay among the women who had a vaginal delivery were third generation cephalosporins (J01DD), which were prescribed in 35% of the cases. This was followed by a combinations of antibacterials (J01RA) prescribed in 20% of the cases and penicillins with extended spectrum (J01CA) prescribed in 15% of the cases. Among the women who had a cesarean section, the most commonly prescribed antibiotic during the stay in hospital was third-generation cephalosporins (J01DD) prescribed in 31% of the cases, followed by the fixed dose combinations of antibacterials (J01RA) prescribed in 30% of the cases and fluoroquinolones (J01MA) prescribed in 13% of the cases. The total DDD/100 bed days during hospital stay for the group of women that had a vaginal delivery was 101 and 127 for women having had a cesarean section (Table 3).

**Table 3:** Description of antibiotics prescribed during hospital stay and at discharge (N = 4721)

		**Vaginal Delivery**			**Cesarean Section**		
		**N (%)**	**Total DDD**	**DDD/100 bed days**	**N (%)**	**Total DDD**	**DDD/100 bed days**
During hospital stay							
ATC	Name						
Total		1496 (100)	1510	101	3225 (100)	4109	127
J01CA	Penicillins with extended spectrum	226 (1)	140	62	46 (1)	35	77
J01CR	Combinations of penicillins, incl beta-lactamase inhibitors	109 (7)	144	132	272 (8)	440	162
J01DB	1^st^ generation cephalosporins	35 (2)	14	41	44 (1)	23	51
J01DC	2^nd^ generation cephalosporins	82 (5)	91	111	116 (4)	135	116
J01DD	3^rd^ generation cephalosporins	525 (35)	628	120	988 (31)	1460	148
J01DH	Carbapenems	4 (0.27)	2	50	3 (0.09)	3	100
J01EE	Combinations of sulfonamides and trimethoprim, incl derivatives	1 (0.07)	0	0	4 (0.12)	0.5	13
J01FA	Macrolides	8 (0.53)	15	188	10 (0.31)	33	333
J01GB	Other aminoglycosides	31 (2.)	18	59	233 (7)	199	86
J01MA	Fluoroquinolones	173 (12)	214	124	430 (13)	709	165
J01RA	Combinations of antibacterials	297 (20)	240	81	983 (30)	1013	103
J01XD	Imidazole derivatives	5 (0.33)	3	60	96 (3)	58	60
At discharge							
Total		425 (100)	151		952 (100)	153	
J01CA	Penicillins with extended spectrum	61 (14)	16				
J01CR	Combinations of penicillins, incl beta-lactamase inhibitors	18 (4)	4		82 (9)	14	
J01DB	1^st^ generation cephalosporins	25 (6)	3		35 (4)	2	
J01DC	2^nd^ generation cephalosporins	66 (16)	32		82 (9)	12	
J01DD	3^rd^ generation cephalosporins	42 (10)	16		104 (11)	26	
J01EE	Combinations of sulfonamides and trimethoprim, incl derivatives	2 (0.47)	0.1		46 (5)	0.5	
J01FA	Macrolides	5 (1)	2				
J01MA	Fluoroquinolones	177 (42)	75		561 (59)	96	
J01RA	Combinations of antibacterials	29 (7)	3.2		42 (4)	2	

Twenty-eight percent of the women with both vaginal deliveries and with cesarean sections were prescribed antibiotics at discharge. The most commonly prescribed group of antibiotic at discharge for women that had a vaginal delivery was fluoroquinolones (J01MA) prescribed in 42% of the cases, followed by second-generation cephalosporins (J01DC) prescribed in 16% of the cases and penicillins with extended spectrum (J01CA) prescribed in 14% of the cases. Among the women that had a cesarean section the most commonly prescribed antibiotics at discharge was fluoroquinolones (J01MA) prescribed in 59% of the cases, followed by third-generation cephalosporins (J01DC) prescribed in 11% of the cases and second-generation cephalosporins (J01DC) and combinations of penicillins, incl beta-lactamase inhibitors (J01CR) prescribed in 9% of the cases each.

## Discussion

Emerging antibiotic resistance is a major global public health challenge. At the same time, untreated infections are one of the main causes of maternal mortality in low and middle-income countries [16]. In India, institutional deliveries are being advocated to reduce the high burden of maternal mortality and morbidity. Increased access to basic and comprehensive emergency obstetric care through the practice of routine institutional deliveries can save the lives of many women, but increased use of antibiotics can also add to the progressing antibiotic resistance in India.

The topic of antibiotic use is not simple in the context of India. It is likely that there is a widespread overuse of antibiotics but also a challenge of antibiotics being unavailable when needed. Access to lifesaving antibiotics is likely to be related to structural determinants of health. The society is stratified by social determinants such as economic status, caste and gender. Women from poor socioeconomic households are less likely to have an institutional delivery or to receive postpartum care compared to women belonging to a household with higher socioeconomic status [17]. Women belonging to these vulnerable groups are likely to be the main beneficiaries of policy on increased coverage of institutional deliveries. However, the same women are likely to be the first to suffer from an increase in antibiotic resistance. New antibiotics needed to meet the challenge of resistance are often more expensive than the predecessor and this is likely to increase the challenge for vulnerable groups to afford treatment with antibiotics in a setting where most maternal health care is paid out-of-pocket [18].

The results from this study show high rates of antibiotic prescribing for both women that had a vaginal delivery and women that had a cesarean section. In the group of women who had a vaginal delivery 491 women (87%) were prescribed antibiotics and in the group of women who had a cesarean section 503 (98%t) were prescribed antibiotics. As a comparison, resent figures from the Christian Medical College (CMC) in Vellore, Tamil Nadu, India showed that among women with a vaginal delivery 22% were prescribed antibiotics and among women having a cesarean section the most commonly prescribed antibiotic was a single dose of cefazolin (Prof S. Chandy, Christian Medical College, Vellore, personal communication). The CMC, Vellore is, in resemblance with the VD Gardi Charitable Trust Hospital, a non-teaching, tertiary hospital that caters both rural and urban population. One difference though is that the CMC, has a general policy on antibiotic prescribing and an active implementation of the policy since long. This indicates that having a policy on antibiotic prescribing and an active implementation of the policy can have an impact on how antibiotics are prescribed also during delivery in hospitals, and can serve as an inspiration for other hospitals in the Indian setting to develop and implement such policy.

Women undergoing cesarean section have a five to 20-fold greater chance of getting an infection compared to women who give birth vaginally, and the routine use of antibiotics at cesarean section reduces the risk of infection [19]. In cesarean section, post-operative infections are likely to be caused by Staphylococcus-epidermidis, Staphylococcus aureus, Group B Streptococci or Enterococcus. The result from the study, showing that 98% of the women having a cesarean section were prescribed antibiotics are therefore not surprising and in line with WHO recommendation. What the study shows, however, is that in the setting studied the type and dose of the prescribed antibiotics differs from the recommendations made by the WHO. The WHO recommends a single dose of cefazoline, a first generation cephalosporin [20,21]. In the setting studied, third generation cephalosporins (J01DD) were prescribed in 30% of the cases, followed by combinations of antibiotics (J01RA) prescribed in 30% of the cases, and fluoroquinolones (J01MA) prescribed in 13% of the cases. Only 2% were prescribed first generation cephalosporins. Data collected did not allow analysis of when prophylactic antibiotics was prescribed but the findings show that the mean number of days on antibiotics prescribed during the hospital stay for women with cesarean sections where 6.0 (±2.5), compared to a single dose recommended by the WHO [22]. Additional studies are needed to better understand the factors influencing the choice of type and duration of prophylactic antibiotics during cesarean sections in this setting.

High levels of antibiotics prescribed to women having a vaginal delivery, both during hospital stay (87%) and at discharge (28%), indicate that antibiotics are prescribed for prophylactic purposes. Prophylactic use of antibiotics during vaginal deliveries in the study setting is not well understood and is beyond the scope of the study presented here. Studies from other settings show that inappropriate prescribing cannot always be explained by lack information and/or knowledge. For example, a study conducted in Lima showed a wide spread practice among physicians to prescribe antibiotics for conditions that did not require treatment with antibiotics despite having good knowledge in terms of appropriate prescribing practices [23]. Heavy workload, lack of information and feeling of pressure to prescribe have been suggested to influence the prescribing of antibiotics [24]. In the Indian setting factor such as varied perceptions of the prescribers, distrust towards the septic conditions of the health facilities and lack of proper understanding of the maintaining asepsis might contribute to high levels of prophylactic prescribing of antibiotics. The factor influencing the choice of, on a routine base, prescribe antibiotics for prophylactic purposes in the setting of India needs to be further studied.

Assisted vaginal delivery is reported to increase the incidence of postpartum infections [25-27]. To reduce the risk of postpartum infections after an assisted vaginal delivery, prophylactic antibiotics are often prescribed. However, the benefits of such practice are not well studied. The few studies available have shown results that both support [28,29] and dismiss [30,31] the use of antibiotics for prophylactic purposes and a Cochrane review on this topic concludes that there are not enough evidence to support the use of antibiotic prophylaxis for operative vaginal delivery but that this needs to be carefully evaluated further [32]. The data collected for this study does not provide information on how many of the conducted vaginal deliveries that were assisted and future studies needs to address assisted vaginal delivery in addition to normal vaginal delivery and cesarean section.

### Methodological considerations

One of the strength of this study is the detailed record of prescribing data on individual patients throughout their hospital stay. In addition, the data includes discharge prescription. The data collection process, with data collected daily by trained hospital staff, is an additional strength. The topic of this study was multifaceted and for this purpose the composition of the group of researchers included competence in drug use, obstetrics, statistics and policy science. The lack of data on socioeconomic status limits the possibilities of comparing antibiotic prescriptions between different economic and social classes. Lack of information on proportion of the assisted or non-assisted vaginal deliveries is a further weakness of the study.

## Conclusions and Policy implications

High percentage of prescribing antibiotics in the patients having vaginal delivery and deviation from the recommendation for cesarean section in the hospital is a cause of concern. The wide spread prescribing of antibiotic to women having vaginal delivery also indicates that antibiotics in this setting is routinely prescribed for prophylactic purposes to women having both normal as well as operative vaginal delivery. This practice needs to be further studied, both in terms of the benefits of prophylactic prescribing during assisted vaginal deliveries and in terms of perceived benefits among health personnel of prescribing antibiotics during non-assisted vaginal delivery.

Improved maternal health and rational use of antibiotics are intertwined. The government of India is advocating for institutional delivery as a strategy to reduce maternal mortality. Following this strategy, there will be a considerable increase in the number of inpatients in the hospitals; this emphasizes the need of a specific policy on how and when to prescribe antibiotics during and after delivery in healthcare facilities. Additionally, monitoring system of antibiotic prescribing and resistance needs to be developed and implemented. As in most cases of policy development and implementation, several interventions are intertwined with each other. Policy on prescribing of antibiotics needs to be linked to policy on interventions on e.g. infection control such as hand hygiene and strengthening postpartum care where a large proportion of infections occur.

## Abbreviations

ATC-Code: The Anatomical Therapeutic Chemical Code; DDD: Defined Daily Dose; MDG: Millennium Development Goal; MMR: Maternal Mortality Rate.

## Competing interest

The authors declare that they have no competing interests.

## Authors’ contribution

MS, KM, SPD and CSL participated in designing the study. MS trained the nursing staff for the data collection and has reviewed the data. MS and KM was responsible for the supervision of the data collection. LS and MS contributed to the statistical analyses of the data. LS and MS has drafted the manuscript. All authors contributed to analyse the results, revised the manuscript critically and approved the final version.
